# Supplementary material for: Earlier emergence of a temperature response to mitigation by filtering annual variability
Source: Nat Commun. 2022 Mar 24;13:1578. doi: 10.1038/s41467-022-29247-y (PMC8948247; doi:10.1038/s41467-022-29247-y)
Supplement: Supplementary file 1 — Supplementary Information [file 41467_2022_29247_MOESM1_ESM.pdf]

Earlier emergence of a temperature response to mitigation by filtering annual variability

B. H. Samset, C. Zhou, J. S. Fuglestvedt, M. T. Lund, J. Marotzke, M. D. Zelinka

Supplementary materials

Monthly resolved Green's Function

For this analysis, we use a monthly resolved Green's function (originally presented in [1]) based on 42 year (2 years spinup + 40 years integration) fixed SST simulations with the CESM1 Earth System Model (see main text). The relation between Global Mean Temperature Anomaly and a one-degree Celsius change at a given ocean location, for a given month, is shown in Supplementary Figure 1.

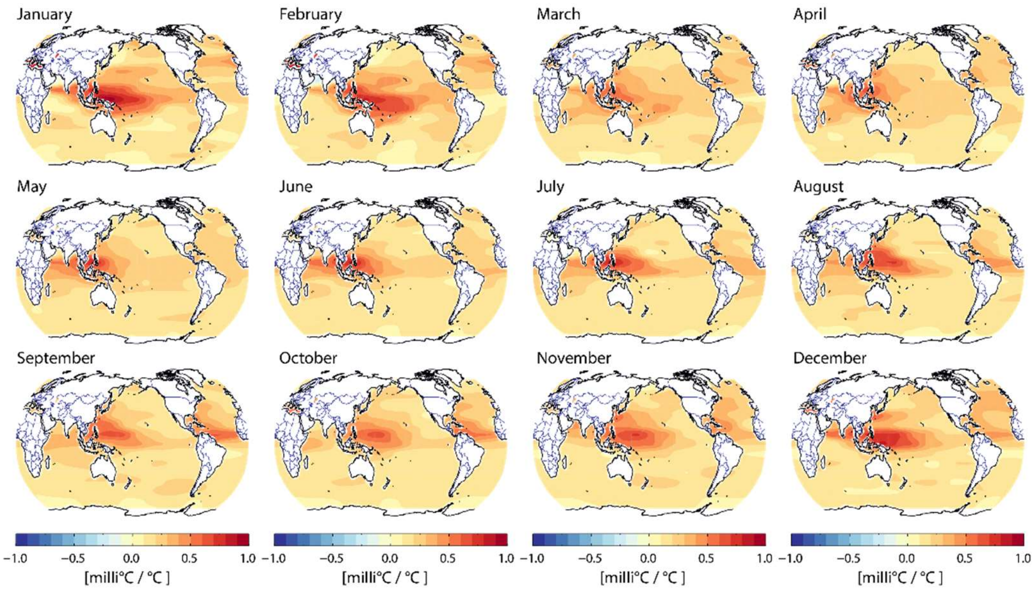

Supplementary Figure 1: Monthly resolved Green's function, linking a change in Sea Surface Temperature in one location to an effect on the Global Surface Temperature Anomaly.

GF filtering performance validation using a 10-member initial condition ensemble

A key question is whether the GF based filtering method moves the GSTA time series closer to a forced response. To test this, we first calculated the ensemble mean GSTA evolution from 10 members from the MPI-ESM1-2 global climate model, running with historical and SSP future emissions (SSP5-8.5, SSP3-7.0, SSP2-4.5, SSP1-2.6; see Methods). We then calculated the standard deviation of the residual of each ensemble member (annual temperature minus multi-ensemble mean temperature), for the filtered and unfiltered cases. See Supplementary Figure 2, which shows how this residual standard deviation is strongly reduced in all cases, indicating that the GF based filtering method does indeed more closely represent the forced response in a dynamically evolving climate.

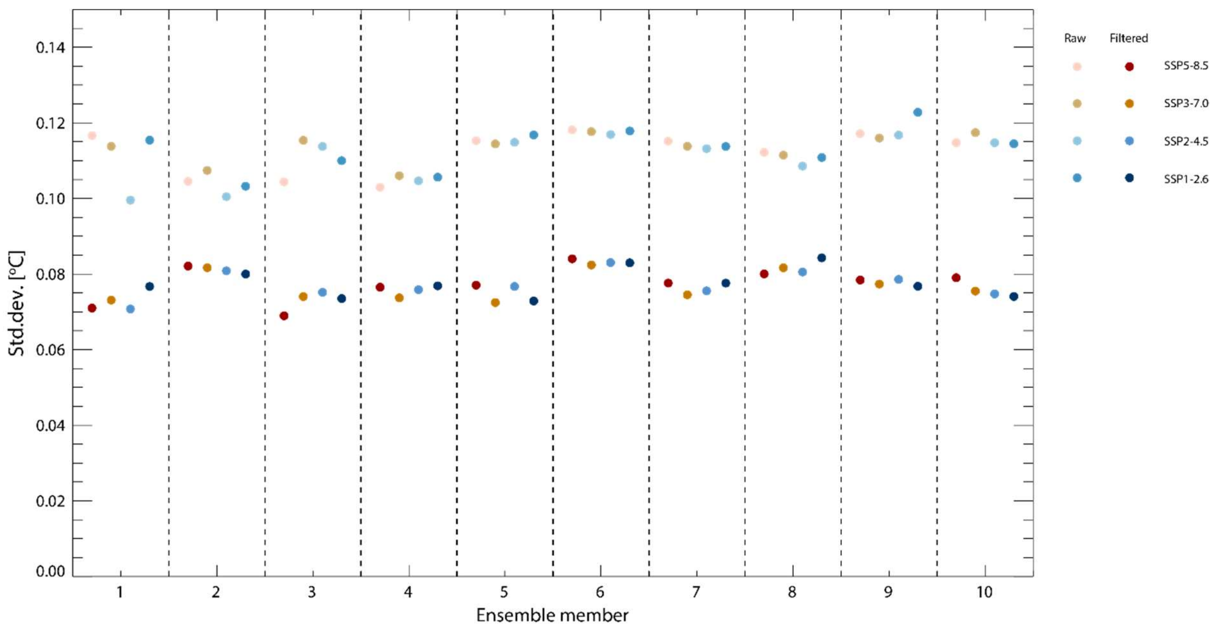

Supplementary Figure 2: Standard deviation of the difference between annual mean GSTA values in a single ensemble member, and the 10-member ensemble mean. Light colors: Unfiltered. Dark colors: Filtered.

Evolution of noise reduction with offset month

In the main analysis presented here, the GSTA modulation is applied to the same month as the generating SST pattern, which implicitly makes the assumption that a majority of the atmospheric response occurs within that time. To test this assumption, we applied the correction also to subsequent months, and quantified the efficiency of the filtering as the standard deviation of the residual relative to a 10-year boxcar average of the unfiltered HadCRUT5 GSTA values. See Supplementary Figure 3, which shows how the standard deviation increases monotonically with offset month, until it approximately equals the raw value after 10 months.

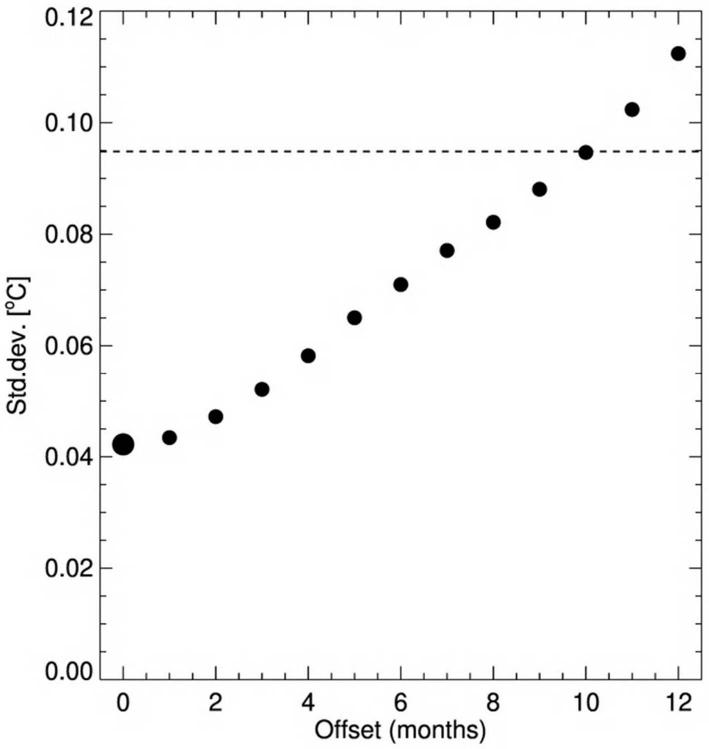

Supplementary Figure 3: The efficiency of the GF filtering as function of the month in which the modulation is applied. The 0 value shows the main analysis. The dashed line shows the unfiltered result.

CMIP6 models used in this study, and the number of realizations from each model

ACCESS-CM2 (1), ACCESS-ESM1-5 (1), AWI-CM-1-1-MR (1), BCC-CSM2-MR (1), CESM2-WACCM (1), CESM2 (1), CNRM-CM6-1-HR (1), CNRM-CM6-1 (1), CNRM-ESM2-1 (1), CanESM5 (20), EC-Earth3-Veg (3), EC-Earth3 (4), FGOALS-f3-L (1), FIO-ESM-2-0 (1), GFDL-ESM4 (1), HadGEM3-GC31-LL (1)m HadGEM3-GC31-MM (1), INM-CM4-8 (1), INM-CM5-0 (1), IPSL-CM6A-LR (6), KACE-1-0-G (2), MCM-UA-1-0 (1), MIROC-ES2L (1), MIROC6 (1), MPI-ESM1-2-HR (1), MPI-ESM1-2-LR (1), MRI-ESM2-0 (1), NESM3 (1),NorESM2-LM (1), NorESM2-MM (1), UKESM1-0-LL (1)

Supplementary References

1 Zhou, C., Lu, J., Hu, Y. & Zelinka, M. D. Responses of the Hadley Circulation to Regional Sea Surface Temperature Changes. *Journal of Climate* **33**, 429-441, doi:10.1175/jcli-d-19-0315.1 (2020).
